# Diagnostic insights into a case of pigmented extramammary Paget’s disease mimicking melanoma^؆^

**DOI:** 10.1016/j.abd.2026.501420

**Published:** 2026-08-10

**Authors:** Melpomeni Theofili, Michael Sofopoulos, Rolandos Ninis, Clio Dessinioti, Irene Stefanaki, Aggeliki Befon

**Affiliations:** a First Department of Dermatology and Venereology, School of Medicine, Andreas Syggros Hospital of Venereal & Skin Diseases, Athens, National Kapodistrian University of Athens, Athens, Greece; b Department of Pathology, Andreas Syggros Hospital of Venereal & Skin Diseases, Athens, Greece; c Department of Surgery, Andreas Syggros Hospital of Venereal & Skin Diseases, Athens, Greece; d State Department of Dermatology and Venereology, Andreas Syggros Hospital of Venereal & Skin Diseases, Athens, Greece

Dear Editor,

We would like to highlight an interesting and diagnostically challenging case of Pigmented Extramammary Paget’s Disease (PEMPD), a rare intraepithelial adenocarcinoma that arises in areas rich in apocrine sweat glands, such as the perineum, the vulva, and, less commonly, the axilla. The pigmentation of PEMPD is attributed to dendritic melanocytes surrounding Paget cells, which endocytose melanin, resulting in clinical and dermoscopic features that closely resemble melanoma.^1^

We report the case of a 56-year-old man with an asymptomatic, flat, ill-defined, dark brown lesion in the suprapubic region that had slowly and progressively enlarged, without evidence of regional lymphadenopathy (Fig. 1). Dermoscopic examination demonstrated features suggestive of melanoma, including white scar-like areas, gray-blue regression structures, and blotches (Fig. 2).

**Fig. 1:**
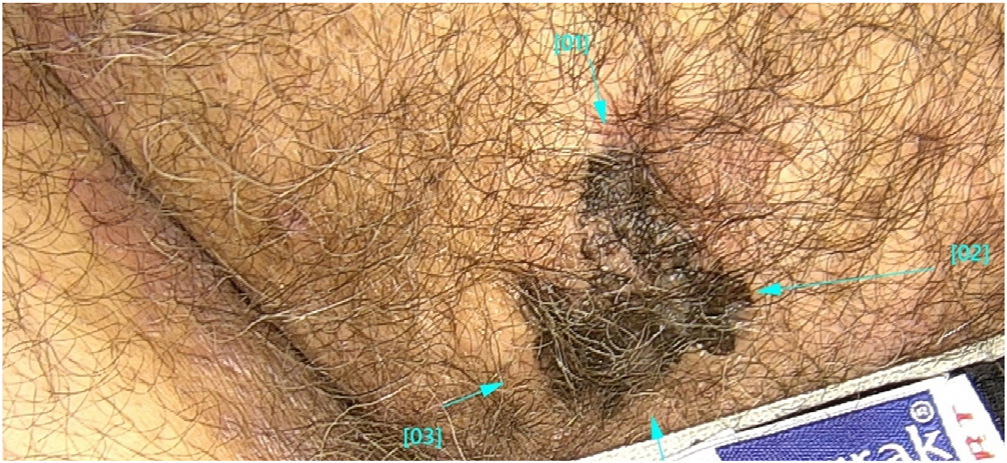
Clinical picture showing a flat, ill-defined, dark brown lesion located in the genital region.

**Fig. 2:**
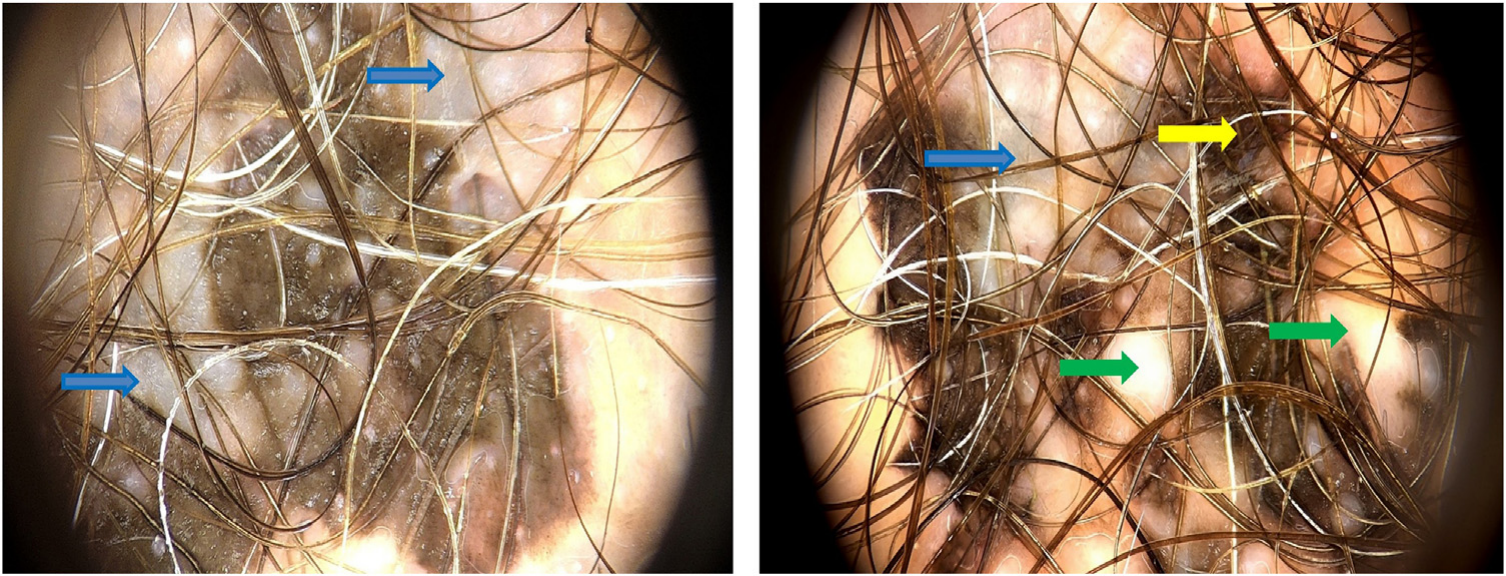
Dermoscopy (20× magnification; FotoFinder digital dermoscopy system) showing white scar-like areas (green arrows), gray-blue regression structures (blue arrows), and a blotch (yellow arrow).

Histopathological examination of the lesion with hematoxylin and eosin (H&E) staining displayed atypical cells within the epidermis. These cells were large and vacuolated, with pale cytoplasm, hyperchromatic nuclei, prominent nucleoli, and some mitoses. They appeared singly or in small clusters (oligocellular nests) surrounded by dendritic cells. Moreover, a mild inflammatory infiltrate was observed in the papillary dermis (Fig. 3). Considering these findings, PEMPD was suspected, and additional immunohistochemical and histochemical tests were used to confirm the diagnosis.

**Fig. 3:**
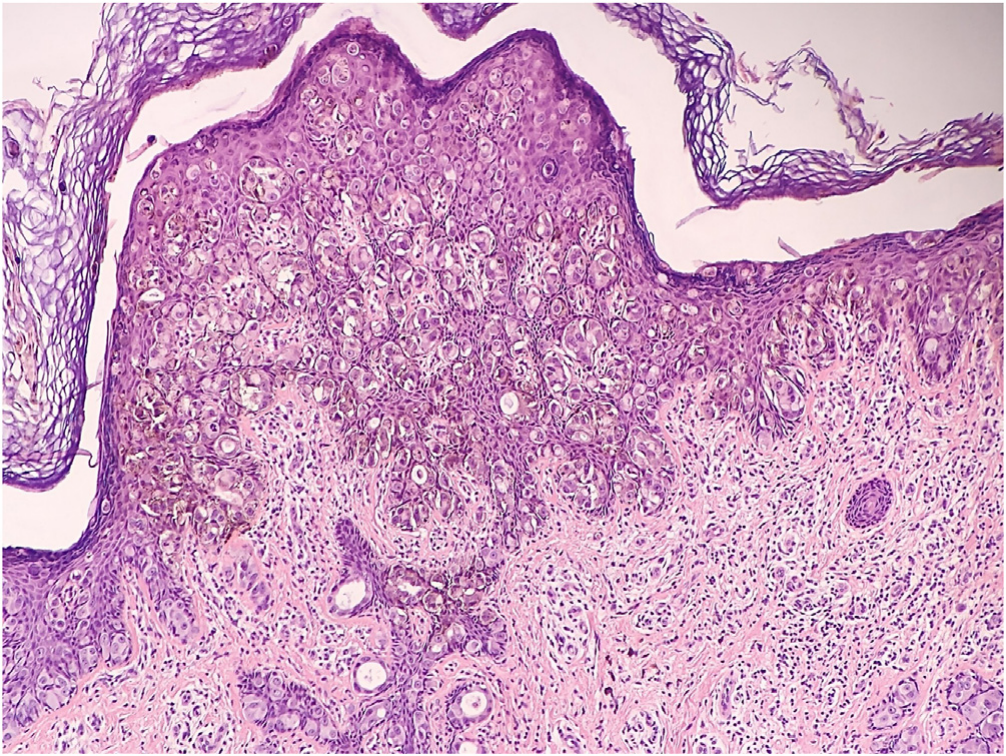
Histopathological image revealing large, vacuolated atypical cells with pale cytoplasm, hyperchromatic nuclei, prominent nucleoli, and occasional mitotic figures spreading in the epidermis in a pagetoid pattern. Dendritic melanocytes are seen interspersed among these cells and focal inflammatory infiltrate admixed with melanophages in the dermis (Hematoxylin & eosin, ×100).

Initially, immunohistochemical examination was performed with Cytokeratin-7 (CK7) and Sex-determining Region Y box transcription factor-10 (SOX10). CK7 is a marker of epithelial cells, employed in the diagnosis of Paget’s disease.^2^ In this case, CK7 stained the atypical pale cells, confirming their epithelial origin (Fig. 4A). Additionally, CK7 staining positivity helps exclude other pigmented lesions, such as malignant melanoma, pigmented squamous cell carcinoma in situ (pigmented Bowen’s disease), and pigmented sebaceous carcinoma, which are negative for CK7 staining.^1^ SOX10, a specific nuclear marker of melanocytes, is used to evaluate melanocytic proliferation and to distinguish melanocytic lesions such as melanoma from epithelial neoplasms.^3^ A slight increase of SOX10-positive melanocytes was identified without evidence of malignant transformation (Fig. 4B). The combination of CK7 positivity in the atypical pale cells and SOX10 positivity confined to mildly increased benign melanocytes supported the diagnosis of PEMPD and excluded melanoma.

**Fig. 4:**
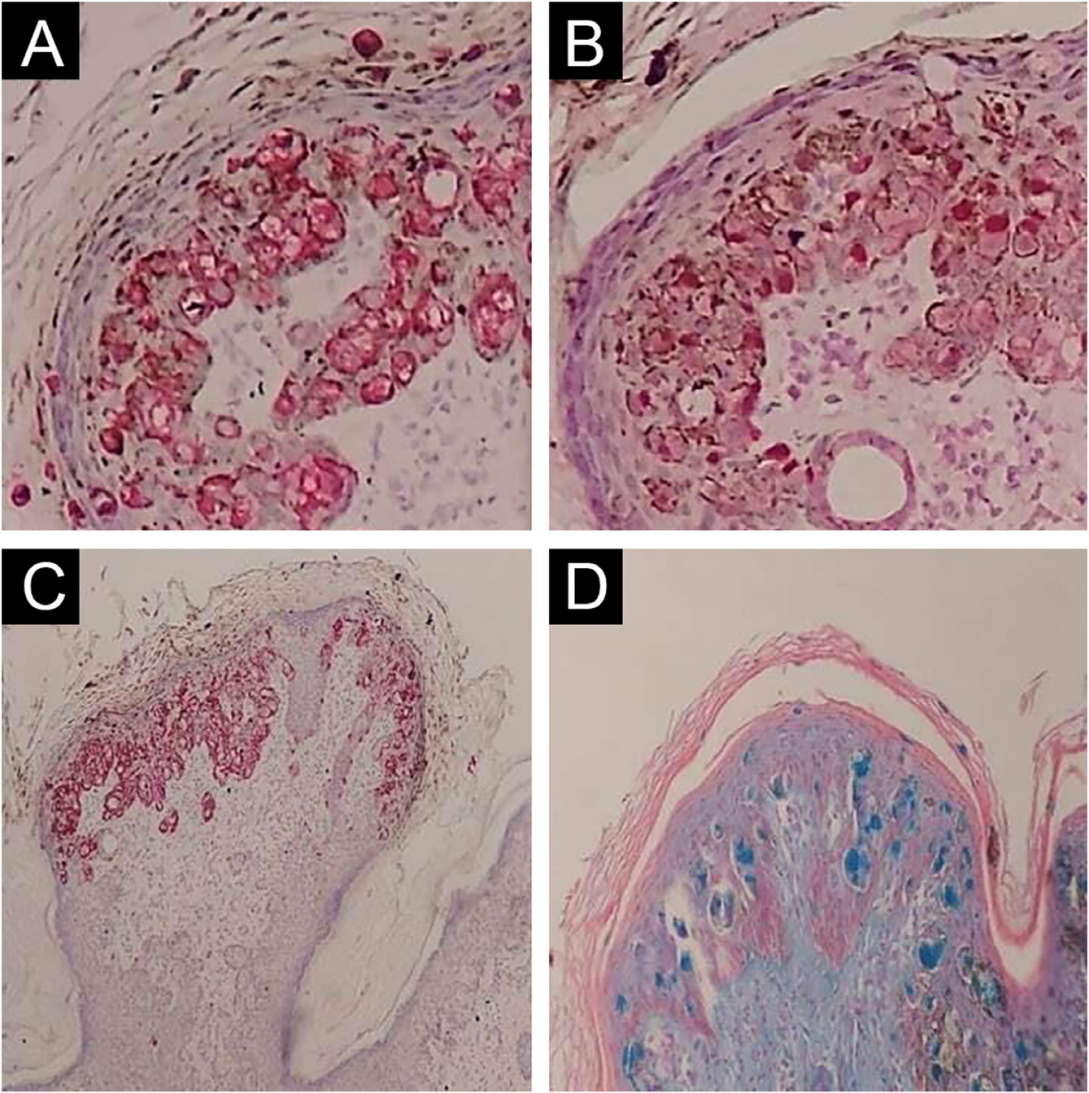
(A) Immunohistochemical staining for CK7 (Cytokeratin 7, ×100) showing cytoplasmic positivity in the atypical cells, confirming their epithelial origin; (B) Immunohistochemical staining for SOX10 (Sex-determining Region Y box transcription factor-10, ×200) demonstrating a mild increase in melanocytes’ nuclei; (C) Immunohistochemical staining for Melan-A (Melanoma Antigen, ×100) demonstrating dendritic melanocytes surrounding nests of atypical epithelial cells; (D) Positive Alcian Blue staining (×100) highlighting the presence of intracytoplasmic mucin within the atypical cells.

To further characterize the melanocytes, Melanoma Antigen (Melan-A or MART-1) immunostain was used. Melan-A-stained melanosomes within the cytoplasm of the melanocytes and delineated their dendritic processes, allowing a detailed visualization of melanocyte distribution. Melan-A-positive melanocytes, with dendritic extensions, were observed surrounding the nests of Paget cells (Fig. 4C).^4^ To clarify the nature of these melanocytes, Preferentially Expressed Antigen in Melanoma (PRAME) immunostaining was carried out. As PRAME is typically expressed in malignant melanocytes, its absence in this case suggests that the surrounding melanocytes were benign.^5^ Final confirmation of the diagnosis was obtained using Alcian Blue staining, which demonstrated the presence of intracellular mucin, reinforced the apocrine primary origin of the tumor, and further ruled out melanoma, pigmented Bowen’s disease, and pigmented sebaceous carcinomas (Fig. 4D).^2^, ^6^ Periodic Acid-Schiff (PAS) staining could also be used to assist in distinguishing Paget’s disease from melanoma, as it highlights intracellular mucin within Paget cells. Considering the histopathological and immunohistochemical findings, a diagnosis of primary PEMPD was made. The patient subsequently underwent a wide local excision. Microscopic evaluation of the specimen revealed clear margins, confirming complete removal of the lesion.

To our knowledge, this is the first described case of PEMPD in the suprapubic area. A review of 23 reported PEMPD cases revealed only six in males, confirming the rarity in men, with two involving the axilla and three the genital region, and two being secondary to prostatic carcinoma. Definitive diagnosis in all reported cases required histochemical stains highlighting Paget cells, melanocytes, and mucin to distinguish PEMPD from other pigmented neoplasms.^2^, ^7^, ^8^, ^9^, ^10^

In conclusion, PEMPD and melanoma share overlapping clinical and dermoscopic features, and routine histopathological examination with H&E staining alone may be insufficient for definitive diagnosis. The use of immunohistochemical markers such as CK7, SOX10, Melan-A, and PRAME, in combination with Alcian Blue staining, enhances diagnostic accuracy.

## Authors' contributions

Aggeliki Befon: Conceived and designed the study and gave final approval of the manuscript.

Melpomeni Theofili: Collected the data, conducted the literature review, drafted, and edited the manuscript.

Michail Sofopoulos: Participated in the diagnostic management of the case and contributed to the analysis and interpretation of the data.

Rolandos Ninis: Was involved in the therapeutic management of the reported case.

Clio Dessinioti: Critically reviewed the literature and revised the manuscript content.

Irene Stefanaki: Critically reviewed the literature and revised the manuscript content.

## Financial support

The authors received no specific funding for this work.

## Research data availability

Does not apply.

## Conflicts of interest

None declared.
